# Robust Semi-Supervised Traffic Sign Recognition via Self-Training and Weakly-Supervised Learning

**DOI:** 10.3390/s20092684

**Published:** 2020-05-08

**Authors:** Obed Tettey Nartey, Guowu Yang, Sarpong Kwadwo Asare, Jinzhao Wu, Lady Nadia Frempong

**Affiliations:** 1 Big Data Research Center, School of Computer Science and Engineering, University of Electronic Science and Technology of China, Chengdu 611731, China; guowu@uestc.edu.cn; 2 Guangxi Key Laboratory of Hybrid Computation and IC Design Analysis, Guangxi University for Nationalities, Nanning 530006, China; gxmdwjzh@aliyun.com; 3 School of Electronic Science and Engineering, University of Electronic Science and Technology of China, Chengdu 611731, China; sk_asare@std.uestc.edu.cn; 4 The School of Computer Science and Electronic Information, Guangxi University, Nanning 530004, China; frempongladynadia@yahoo.com

**Keywords:** traffic sign recognition, semi-supervised learning, self-training, self-paced learning, weakly-supervised learning, deep convolutional neural networks

## Abstract

Traffic sign recognition is a classification problem that poses challenges for computer vision and machine learning algorithms. Although both computer vision and machine learning techniques have constantly been improved to solve this problem, the sudden rise in the number of unlabeled traffic signs has become even more challenging. Large data collation and labeling are tedious and expensive tasks that demand much time, expert knowledge, and fiscal resources to satisfy the hunger of deep neural networks. Aside from that, the problem of having unbalanced data also poses a greater challenge to computer vision and machine learning algorithms to achieve better performance. These problems raise the need to develop algorithms that can fully exploit a large amount of unlabeled data, use a small amount of labeled samples, and be robust to data imbalance to build an efficient and high-quality classifier. In this work, we propose a novel semi-supervised classification technique that is robust to small and unbalanced data. The framework integrates weakly-supervised learning and self-training with self-paced learning to generate attention maps to augment the training set and utilizes a novel pseudo-label generation and selection algorithm to generate and select pseudo-labeled samples. The method improves the performance by: (1) normalizing the class-wise confidence levels to prevent the model from ignoring hard-to-learn samples, thereby solving the imbalanced data problem; (2) jointly learning a model and optimizing pseudo-labels generated on unlabeled data; and (3) enlarging the training set to satisfy the hunger of deep learning models. Extensive evaluations on two public traffic sign recognition datasets demonstrate the effectiveness of the proposed technique and provide a potential solution for practical applications.

## 1. Introduction

**Figure 1:**
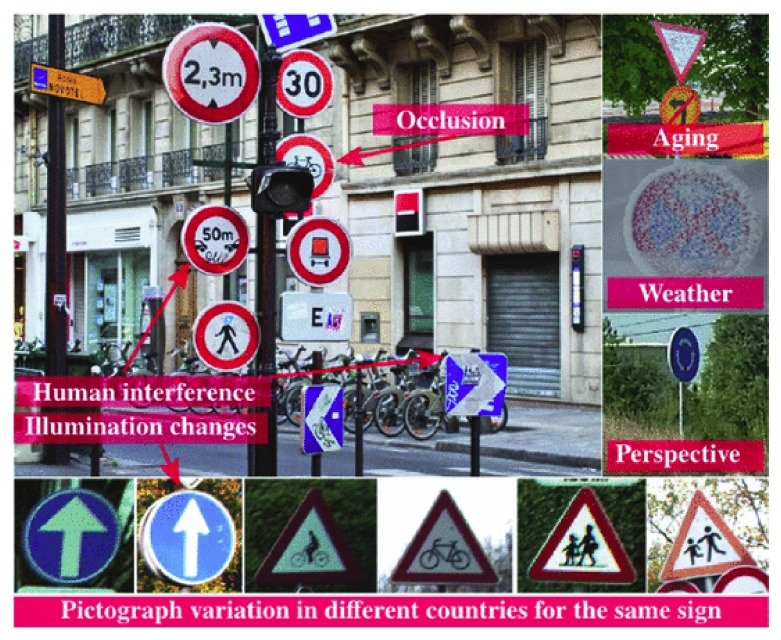
Classification challenges of traffic signs. The signs in the figure are similar to the ones in the GTSRB and BTSC datasets. The image is provided in the book Guide to Convolutional Neural Networks: A Practical Application to Traffic-Sign Detection and Classification [2].

Traffic signs provide reliable safety precautions and guiding information to road users on highways, motorways, urban surroundings, and the sort. In the wake of building smart cities and self-driving vehicles, traffic sign recognition has become a very necessary sub-field of study under object recognition with several applications being developed. Although, many methods have been proposed, still there are issues such as variations in view points, distortion in color of signs especially at night under street lights, blurring from motion, a degradation in contrast, varied poses, and either less or more exposed signs, as depicted in Figure 1, making it difficult to obtain high classification and recognition accuracy. Most methods that have been deployed for traffic sign recognition, be it traditional computer vision methods or advanced ones, have used a supervised learning approach. Classical supervised learning demands all samples to be well annotated before a good model can be built, which is a major drawback when factors such as labeling cost, time, and demand for expertise knowledge are considered. To reduce the labeling cost and make use of both labeled and unlabeled data, a semi-supervised learning technique is used. Semi-supervised learning is an approach that automatically assigns a class to unlabeled samples by relying on its capabilities of predicting labels correctly and through training, extending its knowledge on the predictions learned and/or its competence in classifying [1].

To this end, there is the assumption that less traffic signs that have been well labeled are available together with a chunk of unlabeled ones. Several works [3,4,5,6,7,8] have been done in the past to address the traffic sign recognition task. However, the availability of unlabeled datasets has been less considered and less exploited in terms of traffic sign recognition tasks. With the little literature existing on the topic of Traffic Sign Recognition (TSR) [2,4,6,7,8], it is generally difficult to decide which Convolutional Neural Network (CNN)-based method gives the best result due to the performances that have been reported on benchmark datasets. Some studies [9,10,11] have evaluated their methods on self-gathered private datasets, whereas in other studies [12], the benchmark datasets were combined with self-collated traffic signs, to enrich the dataset for detection and recognition tasks. However, one common thing that is observed among these methods is that they focus on using classical supervised learning to either detect or classify only well-annotated data samples, which results in underperformance when implemented in a real-world scenario. Some of these methods were implemented via traditional hand-crafted features, such as the histogram of oriented gradients (HOG) [11,13,14,15], local binary patterns (LBP) [11], and integral channel features or scale-invariant feature transform (SIFT) [11,16], together with a wide range of machine learning and statistical learning algorithms [9,11,12,14,15,16,17]. Just like in the computer vision world, CNN-based models have been implemented and evaluated in traffic sign recognition tasks. Deep CNNs have achieved huge success in computer vision tasks, cutting across object detection [18,19,20], clustering and association [21], classification [22,23,24], and segmentation [18,25,26]. CNNs have been deployed in many studies [6,7,11,27,28] to learn representations and classifiers automatically. Domen et al. [28] proposed a deep learning framework with end-to-end full feature learning. Their approach was based on Mask R-CNN, which used a region proposal network to employ deeper network architectures in detecting and classifying traffic signs. In the study [29] conducted by Alvaro et al., a single CNN for automatic recognition of traffic signs that alternated convolutional and spatial transformer modules was utilized. Extensive experiments were conducted on the German Traffic-Sign Recognition Benchmark (GTSRB) and the Belgium Traffic Sign for Classification (BTSC) dataset to find the best CNN architecture, as well as to investigate the impact of multiple spatial transform network configurations within the CNN, together with the effectiveness of four stochastic gradient decent optimization algorithms. A recognition accuracy of 99.71% was obtained for precision, recall, and F1-score for the GTSRB and the BTSC; 98.95%, 98.87%, and 98.86% were obtained for the precision, recall, and F1-score, respectively. The recognition rate was improved by the study conducted by Mahmoud et al. [30]. Mahmoud et al. combined features learned by deep convolutional generative adversarial networks (DCGAN) and pseudoinverse learning autoencoder (PILAE) supplemented with the softmax classifier method to obtain excellent performance with a recognition rate of 99.80% on the GTSRB and 99.72% on the BTSC, as compared to handcrafted features and other methods that were DNN based. DCGAN was utilized to extract the informative features in an unsupervised way without needing an expert analysis of the learning process and PILAE to train the model faster. Furthermore, Sermanet et al. applied the convolutional network architecture to achieve a better result on the GTSRB dataset after experimenting with the Energy-based learning (EBLearn) open-source library [31]. However, their result of 99.17% was later improved by the work of Mahmoud et al. [30]. Another study proposed Balancing GAN (BAGAN) [32] as an augmentation tool to restore balance in imbalanced datasets. The method generates images for the less represented classes from the majority classes, and during the adversarial training, all available images of the majority and minority classes are included in the training process. The generative model learns useful features from majority classes and uses these to generate images for the minority classes. The study further used class conditioning in the latent space to drive the generation process towards a target class. Competitive and decent results were achieved. However, all these methods, as mentioned previously, are classical fully-supervised learning techniques. In the sub-field of weakly-supervised learning (WSL), object detection and segmentation involve locating and segmenting with image labels [24,26,33]. Object detection problems are solved with weakly-supervised learning as a classification problem by pooling layers in CNN models. In the work [24] conducted by Durand et al., they used a weakly-supervised learning model to learn and localize visual parts that were related to class modalities. They were able to classify images, as well as supervise weakly the pointwise localization of objects and segmentation. Existing weakly-supervised learning methods were improved at three levels, where they made use of fully-convolutional networks (FCNs) as baseline models in their method. They aggregated spatial scores into a global prediction. Wang et al. [34] improved on [24] by using an iterative top-down and bottom-up architecture to expand object regions and also optimize the network. The method was further improved by [35] to mine object locations and pixel labels via filtering and fusion of multiple pieces of evidence. They proposed an algorithm for filtering, fusing, and categorizing object instances collected from multiple solution mechanisms. The method achieved great success and challenged state-of-the-art algorithms. Ge et al. [36] then combined the algorithms proposed in [34,35] using a bottom-up approach weakly-supervised learning to classify fine-grained images. They performed weakly-supervised instance detection and segmentation and proposed regions for Mask R-CNN [37] by using Class Activation Maps (CAM) [13]. They rectified the object regions and masks iteratively with Conditional Random Fields (CRF) [38] as a way to prevent losing significant parts for object’s parts modeling. Given these successes, we adopted weakly-supervised learning to generate attention maps and selected the most important parts from multiple proposed parts in each image in an annotation-free scenario using attention cropping [39]. In this way, we enhanced discriminative feature representation and at the same time captured wide feature parts.

However, one challenge in recent computer vision tasks is how to obtain a large amount of well-annotated data. The labeling challenge originates from two perspectives: First, a large number of labeled samples are required to be able to create a model that will easily generalize and precisely depict a real-world situation for a whole dataset. Secondly, different annotators have semantic gaps. There is no universal standard for the annotation of these samples, so different annotators give different positions for the same data samples. Furthermore, collecting images that capture all possible instances of objects in an ever-changing world is not feasible. Moreover, the burden of annotating is amplified more when we have to consider traffic sign recognition. In this setting, only experts will be able to provide well-labeled data for the recognition model.

Fortunately, through semi-supervised and weakly-supervised learning, a robust semi-supervised traffic sign recognition can to some extent alleviate the costly and laboriousannotations by utilizing unlabeled images. Techniques such as those in [40,41,42,43,44] use self-training or similar concepts to utilize unlabeled samples for semi-supervised learning. A greedy unsupervised criterion has been used to generate and select the pseudo-labeled data for the retraining process of models. Most of the time, this criterion is the loss of the pseudo-labeled data, where its predicted approximate label is considered as the true label to calculate the loss [41,45]. Since no supervision is required during the retraining procedure and training the criterion function, the loss criterion has a high tendency of producing incorrect pseudo-labels and selecting incorrect pseudo-labeled data for the retraining process. This way, these incorrect pseudo-labeled data mislead the optimization of the classifier and detector with the consequence of reinforcing the wrong data in the unsupervised retraining phase. When it comes to the application of semi-supervised learning methods for the traffic sign recognition task, a few literature works can be found. He et al. proposed a novel semi-supervised learning method that combined global and local features for traffic sign recognition in an Internet of Things-based transport system [46]. In that research, different feature spaces were built utilizing approaches such as the histogram of oriented gradients (HOG), color histograms (CH), and edge features (EF) for the labeled aspect and for the unlabeled data samples. He et al. used the fusion of the feature space to alleviate the differences between the varying feature spaces. By employing a semi-supervised tri-training, a classifier was trained to obtain a 98.7% recognition rate and also to solve a small sample problem. However, the authors failed to tackle the issue of class imbalance, which led to reinforcing incorrectly generated pseudo-labels as a result of the model holding on to the well-represented categories, causing the performance of some classifiers to decline eventually. Hillebrand et al. proposed applying semi-supervised co-training to classify German traffic signs [1] prior to the study by He et al. In that research, Hillebrand et al. deployed an iterative co-training process where the most informative samples from a given pool of unlabeled traffic signs were automatically selected and then classified by two classifiers, which generated labels for each other [1]. Extensive experiments were conducted on 14 classes of German traffic signs to obtain an accuracy of 98.0%, which would later be improved by the work of He et al. [46].

In contrast to the techniques mentioned in the literature, which are mostly supervised learning schemes, we propose Robust Semi-Supervised Traffic Sign Recognition (ROSST). It integrates Weakly-Supervised Learning (WSL) [24] and Self-Paced Learning (SPL) [41] to build a semi-supervised learning model. The WSL technique is used to extract reliable attention maps from images by magnifying the attention parts. Then, it augments those parts of the training samples for the (ROSST) framework, after which the self-training scheme with self-paced learning are used to train a classifier. The SPL framework, in its optimization process, selects “easy” training samples, avoids noisy instances at the initial stages, and gradually learns hard-to-transfer samples. In summary, the main contributions of ROSST are as follows:

- We propose a novel ROSST framework that takes into consideration the challenges of imbalanced datasets by utilizing weakly-supervised learning to generate attention maps representing the spatial distributions of an object’s parts, to extract local features, and via self-paced learning, to solve a small sample problem using the traffic sign recognition problem. To prevent the case of missing any object parts, the proposed regions in the detection phase are further refined by building a complementary model that covers the proposed regions consisting of object information as much as possible. The deep features are then encoded for classification.
- Secondly, we use an easy-to-hard self-paced learning to improve or achieve the high classification accuracy that has been obtained by supervised learning algorithms with just 60% of the annotated training set. The remaining 40% is added to the test set and used as a non-annotated set during pseudo-labeled data generation. In brief, during the training iterations, the chosen pseudo-labeled samples go from “easy” (with relatively high confidence), where the optimization procedure selects “easy” pseudo-labeled training samples and avoids noisy instances, to “hard” (rare classes), and it gradually updates the classifier by retraining it with the selected pseudo-labeled samples. This way, the challenge of generating reliable pseudo-labeled samples (with high precision) for the training iteration is ensured. Another challenge of getting enough reliable labeled data for deep CNN models to obtain good accuracy is also tackled by looking for possibly many newly-labeled samples (high recall) where reliable pseudo-labels with high precision are assigned to unlabeled samples for the retraining process.
- To improve the deep neural network’s accuracy on a small amount of annotated data, we combine CNN and self-training by formulating a loss minimization scheme, solving it by using an end-to-end approach to learn domain-invariant features and a classifier. Therefore, we aim to learn the discriminatory features by building a target-specific network and feed it with the artificially labeled samples together with the labeled training set.
- In dealing with the class imbalance problem of pseudo-labels’ generation in self-training methods, we propose confidence scores that utilize class-wise normalization to generate and select pseudo-labels with a balanced class distribution. To achieve this, we develop an effective algorithm to solve the optimization by normalizing the class-wise confidence scores. Experimental results on two benchmark datasets demonstrate the effectiveness and robustness of the proposed method.

The remainder of this paper is organized as follows. Section 2, provides the detailed description of the methods and materials together with how the optimal parameters will be used for the model. Step-by-step descriptions are provided for each component of the proposed method. In Section 3, the description of the data that were used, the network protocol, the training strategies, and the design choices are provided. The experimental results of the different training strategies and a discussion of the results in the context of related works are presented in Section 4, and finally, Section 5 gives the conclusion to the paper.

## 2. Materials and Methods

### 2.1. ROSST Overview

**Figure 2:**
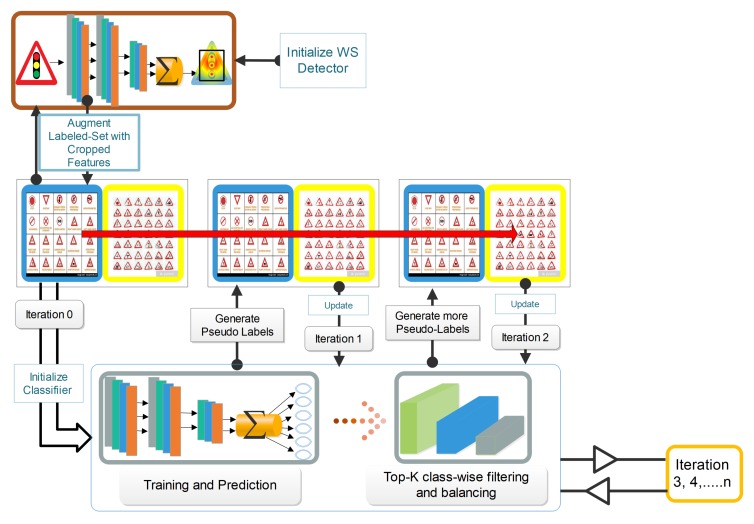
The framework of ROSST. The boxes in the middle row contain the training images where the few labeled and the many unlabeled images are in the blue and yellow areas, respectively. The gray round rectangle represents the classifier, a custom deep learner. We train the classifier using a few annotated images. The weakly-supervised detector in the top row generates reliable informative parts and augments the labeled training set for the classifier to be trained on, as shown as Iteration 1. In the following iterations, the classifier generates reliable pseudo-labels to further update the classifier. The classifier becomes robust when the generation of pseudo-labels and updating of the classifier are iteratively carried out from “easy” to “hard”.

We begin by providing an overview of our approach to classifying traffic signs via self-training, as illustrated in Figure 2, and then elaborate on each of the steps. We combined the Semi-Supervised Learning (SSL) and Weakly-Supervised Learning (WSL) methods to localize and classify traffic signs by training a CNN on a relatively small percentage of images with both strong and weak annotations and a large set of unlabeled traffic sign data. Looking at the workflow of the proposed system illustrated in Figure 2, the framework used a Convolutional Neural Network (CNN)-based self-training model with weakly-supervised learning to detect and learn discriminative feature representations that were compact, which synchronously implemented attention mapping and region proposals for classification by the model with no bounding box annotation. We assumed that we were given a small amount of labeled traffic signs (blue boxes in the middle row of Figure 2) and a huge amount of unlabeled traffic sign data (yellow boxes in the middle row of Figure 2) with instances of the object classes within some of these labeled traffic signs. We initialized a weakly-supervised detector (top row of Figure 2) that was trained with images that had no bounding box annotations, to propose regions from attention maps and to crop and enlarge the localized part to augment labeled samples, which were then used to perform a supervised training (bottom row of Figure 2). In this way, complementary parts and most discriminative parts were mined and augmented in the training set to train the classifier using self-training. We then trained the classifier with 60% of the labeled samples. The trained model was then evaluated on the unlabeled data to generate pseudo-labels for the unlabeled data based on the predictions of the model. A selection algorithm with a class balancing mechanism (bottom row of Figure 2) was then run to select the pseudo-samples that had the highest confidence probability score with their pseudo-labels, on which they were then retrained. We utilized the pseudo-labeled samples to improve and prevent the complicated model tuning for the traffic recognition task to meet or improve the recognition accuracy of the state-of-the-art supervised learning traffic recognition models. The advantages of ROSST were mainly four-fold. First, the method reduced the labeling cost by utilizing only a few annotated images per class. Secondly, ROSST provided robust supervision to rare classes, where only a few training images could be found through a self-paced learning scheme. For such classes, an image-level supervision of the limited number of samples is never enough to train a good learner. Thirdly, ROSST could deal with with tiny images, by enlarging and focusing on the most important informative object parts. Fourth, ROSST provided accurate labels to sample images and obtained high accuracy on a small amount of datasets, while models trained with image-level labels usually performed poorly on the same small amount of data. In comparison, using a few images with annotations, the method could enhance the model to be robust and still produce high and decent classification accuracy on different percentages of labeled data, which was evident from our experimental results, as given in Table 6.

### 2.2. Semi-Supervised Traffic Recognition

The traffic recognition task datasets were much smaller than general-object large visual classification datasets, and like computer vision tasks, traffic sign recognition tasks have recently been subjected to the use of CNNs. A great deal of imbalance across the classes in the traffic recognition task dataset caused a different degree of difficulty in the prediction confidence levels for unlabeled target datasets. Additionally, samples in the various classes were not the true accurate representation of the characteristic differences in the visual classes themselves. In expectation maximization algorithms, one thing that frequently occurs is that the algorithms easily hold on to sample-specific features from classes that have higher samples than classes with few samples, and they are not well represented during the training process. This forces the model to abandon the versatile visual features that need to be learned. Over-fitting is another issue that hinders deep CNN learning models from obtaining higher performances when trained with a small set of data. Deep CNN learning models therefore find it difficult to replicate the performances they obtain at training time when deployed in real-world scenarios. In the presence of such challenges, we followed an “easy experimental settings to hard” procedure via self-paced learning, utilizing a class-wise confidence score normalization in generating and selecting reliable pseudo-labels from the most confident predictions with a balanced class distribution, making sure that the model was well updated and better adapted to the test domain. Compared to other visual classification approaches, the advantages of ROSST as stated previously can be further combined into two: (1) by utilizing a self-paced “easy-to-hard’ curriculum learning, we propose an expectation maximization algorithm via self-training to avoid reinforcing the wrong predictions to enlarge the training set during the training process; that is, to prevent and alleviate the model from retraining and learning from only well-transferred classes, ignoring the “hard” or not well-represented classes along the training procedure; (2) we propose a robust technique that can detect and classify samples from a small labeled sample size and utilize its effectiveness for a more real-world scenario. To this end, this work is the first to tackle the traffic sign recognition problem from a semi-supervised approach that utilizes strongly-labeled, weakly-labeled, and unlabeled data.

### 2.3. Weakly-Supervised Attention Learning

We adopted weakly-supervised learning to localize object parts only by their class annotations. We extracted the feature of labeled image $I_{l}$ by a deep Convolutional Neural Network (CNN) and term $FM\in R^{H\times W\times C}$ as the feature maps, with H,W,andC being the height, width, and channels of the image features, respectively. We obtained the attention map $A\in R^{H\times W\times C}$ from FM as an object’s representation by:

(1) $$Att=f\left(FM\right)=\overset{M}{\underset{m=1}{⋃}}Att_{m}$$

From Equation (1), $f\left(\cdot \right)$ is the convolutional function that takes FM as an input. $Att_{m}\in R^{H\times W}$ is the representation of the object part. It is the attention maps that represent the visual pattern of the object. It could be an inscription, a symbol, or an outline of the traffic sign, and *m* is the number of attention maps; however, for this study, m=1. The attention maps were cropped out and were augmented to the labeled training set. We extracted informative and discriminative feature maps by a feature extracting function g(·). In this case, the extracting function used was a set of convolutions with Global Average Pooling (GAP) to obtain the $m_{th}$ feature part $fm_{m}\in R^{1\times N}$ as shown in Equation (2), and we multiplied it element-wise by each attention map $Att_{m}$.

(2) $$fm_{m}=g\left(FM_{m}\right).$$

To get an object’s feature, several feature parts $fm_{m}$ were stacked to form a part feature matrix PFM. Letting PFM be the local attention pooling that exists between the attention maps $Att_{m}$ and feature maps FM, we represented the object’s feature as:

(3) $$\begin{array}{c} PFM=\left(\begin{array}{c} g(att_{1}•F)\\ g(att_{2}•F)\\ g(att_{3}•F)\\ \cdots \\ g(att_{M}•F) \end{array}\right)=\left(\begin{array}{c} fm_{1}\\ fm_{2}\\ fm_{3}\\ \cdots \\ fm_{M} \end{array}\right) \end{array}$$

**Figure 3:**
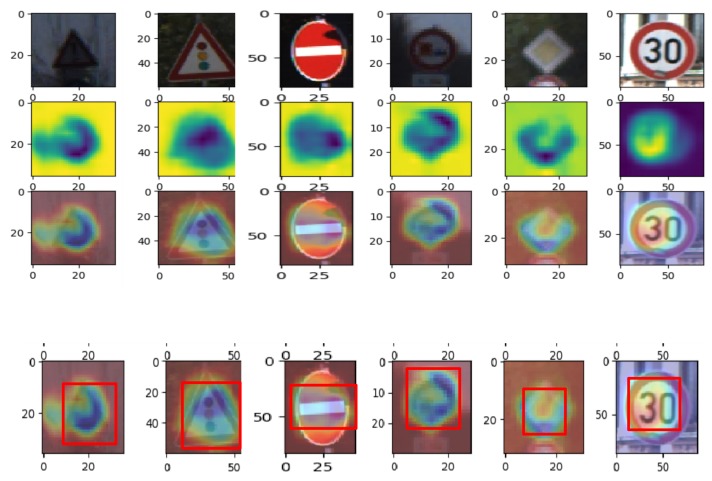
Feature maps: Attention maps represent the discriminative parts of the traffic sign. The image part is localized from the attention maps and enlarged to further improve cropping. The top row is the original traffic signs; the second row contains the activation maps; and the third row shows the part of the original images on which the model is focusing. The fourth row shows the discriminative part to be cropped with the bounding box.

Augmenting the training data by the random cropping technique was less effective, especially when either the image is small or occluded. However, with attention maps, the data can be more augmented with a very high efficiency. By adopting the attention cropping technique proposed by Hu et al. [39], we augmented the labeled training samples by using the attention maps generated. The method initially obtained the crop mask, found a bounding box that covered the selected positive part as depicted in Figure 3, and then, enlarged the selected portion to be used as the input for the augmented data. With this, objects could be seen better by the model by focusing on the cropped informative features, since the scale of the objects increased.

### 2.4. Self-Training Preliminaries

Suppose we have a few *l* labeled images; the most efficient way to utilize a small of amount data and still improve classification accuracy is through supervised fine-tuning of the models. For classification networks with a softmax output having *n* classes, the objective function with data $X_{l},Y_{l}$ can be defined as minimizing the loss:

(4) $${L_{c}\left(X_{l},Y_{l}:\theta_{c}\right)}_{W}=-\sum_{k}^{n}1\left[y=k\right]\log P_{k}$$

In Equation (4), the aim is to build a model $\theta_{c}$ that can correctly classify samples at test time. However, assuming that there is a huge amount of unlabeled data, the transfer of representations using fine-tuning becomes inefficient, and the semi-supervised classification technique has to be used by adapting a trained model on a similar set of data to the unlabeled data. This leads to formulating the problem as minimizing the loss function:

(5) $$\begin{array}{cc} {minL_{c}\left(W,\hat{Y}\right)}_{W}= & -\sum_{l=1}^{L}\sum_{n=1}^{N}Y_{l,n}^{L}\log\left(P_{n}\left(W,I_{l}\right)\right)\\ \; & -\sum_{u=1}^{U}\sum_{n=1}^{N}{\hat{Y}}_{u,n}^{U}\log\left(P_{n}\left(W,I_{u}\right)\right) \end{array}$$

where $I_{l}$ denotes a labeled image in the source domain that is indexed by l=1,2,…,L with its true label $Y_{l,n}$ for the nth image (n=1,2,…,N), *W* indicates the network weights, and the softmax output $P_{n}\left(w,I_{s}\right)$ contains the probabilities for the various classes of the traffic sign dataset. For the unlabeled set, $I_{u}$ denotes the image in the target domain, where the ground truth label is unavailable. ${\hat{Y}}_{u,n}$ is an estimated target label that, when optimized, approximates the true label. At evaluation time, the ground truth label is considered to be a hidden variable that can be learned by minimizing Equation (5). Similarly, $p_{n}\left(w,I_{u}\right)$ is the output of the softmax, which contains the class probabilities for the approximated labels. However, the domain gap of feature spaces between the source and target domains makes it difficult to obtain a generalized model that can perform well in real-world scenarios. In view of this challenge, we propose to learn a model and optimize jointly the labels that would be learned on the unlabeled data in the target domain with several iterations of the training the model; a training technique we would regard as self-training.

### 2.5. Self-Training with Self-Paced Curriculum Learning

It is difficult to learn a model and optimize approximate labels on non-annotated data jointly. Therefore, a better methodology is to follow an “easy-to-hard” plan by utilizing self-paced curriculum learning to generate approximate labels also termed “pseudo-labels” from the “easy” (highest confidence) predictions, trusting that they are mostly accurate and rightly approximate the ground truth labels. Given that the model is well updated and adapted to the unlabeled data in the target domain, the remaining less-confident (hard) pseudo-labels are then looked at and explored. Combining the curriculum learning and the self-training strategy, we modified and formulated the loss function as:

(6) $$\begin{array}{cc} {minL_{c}\left(W,\hat{Y}\right)}_{W,\hat{Y}}= & -\sum_{l=1}^{L}\sum_{n=1}^{N}Y_{l,n}^{L}\log\left(P_{n}\left(W,I_{l}\right)\right)\\ \; & -\sum_{u=1}^{U}\sum_{n=1}^{N}\left[{\hat{Y}}_{u,n}^{U}\log\left(P_{n}\left(W,I_{u}\right)\right)\;+\;k_{c}{\hat{Y}}_{u,n}^{\left(c\right)}\right].\\ \; & s.t.\;{\hat{Y}}_{u,n}\in \left\{e^{\left(i\right)}\in R^{C}\right\},k_{c}>0 \end{array}$$

From Equation (6), *Y* is designated 0, leading to the rejection of the pseudo-label $\hat{Y}$ during the training phase of the model. The hyperparameter $k_{c}$ in Equation (6) is used to control the quantity of pseudo-labeled samples to be selected from the classes *c*. This implies that a large $k_{c}$ ensures that a large amount of pseudo-labeled samples would be selected to update the model. $e^{\left(i\right)}$ is a one-hot encoded vector. Because self-training techniques generate pseudo-labels that correspond to high confidence, one problem that comes up is that the model tends to be biased towards the easy-to-transfer samples. The difference in the visual domain gap and class distribution causes some transfer challenges among classes, resulting in relatively higher prediction accuracy scores for easy-to-transfer target domain samples. The model in such scenarios ignores other hard or not well-represented classes during the training procedure. By introducing $k_{c}$ in Equation (6), a different level of class-wise bias for the selection of pseudo-labels to tackle the problem of class imbalance emerges.

1. Initialize *W*, and minimize the loss in Equation (6) with respect to ${\hat{Y}}_{u,n}$.
2. Set ${\hat{Y}}_{u,n}$, and optimize the objective function in Equation (6) with respect to *W*.

We iteratively alternated the steps of Executing Step 1 followed by Step 2 to minimize the loss in Equation (6). Executing Steps 1 and 2 was considered to be a single iteration or round, and as we proposed a self-training-based algorithm, Steps 1 and 2 were alternatively repeated for several iterations. Executing Step 1 led to the selection of a portion of the most-confident pseudo-labels on the unlabeled dataset, whereas in Step 2, the model was trained given the pseudo-labels chosen in Step 1. Step 2 was solved leading to the probabilistic learning of the model with a gradient decent optimization technique. In solving Step 1, given the optimization over discrete variables, a nonlinear function was required, leading to Step 1 being reformulated as Equation (7), if K>0.

(7) $$\begin{array}{cc} \; & min_{\hat{Y}}-\sum_{u=1}^{U}\sum_{n=1}^{N}\sum_{c=1}^{C}\left[{\hat{Y}}_{u,y}^{\left(c\right)}\log\left(p_{n}\left(C|w,I_{t}\right)\right)+k_{c}{\hat{Y}}_{u,n}^{\left(c\right)}\right].\\ \; & \;s.t.\;{\hat{Y}}_{u,n}=\left[{\hat{Y}}_{u,n}^{\left(1\right)},\ldots ,{\hat{Y}}_{u,n}^{\left(c\right)}\right]\in \left\{e^{\left(i\right)}\in R^{C}\right\},k_{c}>0 \end{array}$$

It can be observed that the formulation in Equation (7) was similar to the work proposed in [44]. However, the difference between the formulation in Equation (7) and the optimization flow in [44] was that we introduced a class-wise bias by normalizing class-wise confidence levels compared to the use of an $L_{1}$ regularizer to prevent most pseudo-labels from being ignored by serving as a negative sparse term. The authors in [44] solved the pseudo-label framework optimizer by utilizing the solver in Equation (8).

(8) $${\hat{Y}}_{u,y}^{\left(c*\right)}=\left\{\begin{array}{cc} 1, & \mathrm{if}\;c=arg maxp_{n}\left(c|w,I_{u}\right),\\ \; & p_{n}\left(c|w,I_{u}\right)>\exp\left(-k\right)\\ 0, & \mathrm{otherwise}. \end{array}\right.$$

Noticeably, the pseudo-label generation and selection were dependent on the output $p_{n}\left(c|w,I_{u}\right)$, which did not explicitly solve the class-imbalance problem that resulted in Expectation Maximization (EM) methods being biased towards easy-to-transfer samples. To tackle the class-imbalance problem, we reformulated Equation (6) as:

(9) $$\begin{array}{cc} {minL_{c}\left(W,\hat{Y}\right)}_{W,\hat{Y}}= & -\sum_{l=1}^{L}\sum_{n=1}^{N}Y_{l,n}^{L}\log\left(P_{n}\left(W,I_{l}\right)\right)\\ \; & -\sum_{u=1}^{U}\sum_{n=1}^{N}\sum_{c=1}^{C}\left[{\hat{Y}}_{u,n}^{U}\log\left(P_{n}\left(W,I_{u}\right)\right)\;+\;k_{c}{\hat{Y}}_{u,n}^{\left(c\right)}\right].\\ \; & \;s.t.\;{\hat{Y}}_{u,n}=\left[{\hat{Y}}_{u,n}^{\left(1\right)},\ldots ,{\hat{Y}}_{u,n}^{\left(c\right)}\right]\in \left\{e^{\left(i\right)}\in R^{C}\right\},k_{c}>0 \end{array}$$

In Equation (9), a normalizer was incorporated into the self-training configuration where class-wise confidence scores would be normalized to tackle the challenge of class balancing. The loss function in Equation (7) was used to minimize the optimization framework in Equation (9), however with a different solver as provided in Equation (10) incorporating the class-wise normalizing term:

(10) $${\hat{Y}}_{u,y}^{\left(c*\right)}=\left\{\begin{array}{cc} 1, & \mathrm{if}\;c=arg max\frac{p_{n}\left(c|w,I_{u}\right)}{\exp(-k_{c})},\\ \; & \frac{p_{n}\left(c|w,I_{u}\right)}{\exp(-k_{c})}>1\\ 0, & \mathrm{otherwise}. \end{array}\right.$$

From Equation (10), the pseudo-label generation and selection were not dependent on the prediction confidence level output as given in the solver by [44]. Instead, this was dependent on the class-wise normalized output $\frac{p_{n}\left(c|w,I_{u}\right)}{\exp(-k_{c})}$. Assigning the pseudo-label to an unlabeled sample by utilizing the normalized output gave the advantage of balancing towards the class that had a relatively low score, but a high within-class confidence score.

### 2.6. Determining the $K_{c}$ Algorithm

$K_{c}$ as stated previously plays a vital role in distilling pseudo-labels’ probability values that are less than $K_{c}$. We set $K_{c}$ using the procedure in Algorithm 1 to control the proportion of pseudo-labeled samples selected and used to update the model in each iteration. The algorithm to determine $K_{c}$ encodes the class-wise confidence levels effectively by ranking the class *C* probabilities on all image samples predicted as class *C*, and we set $K_{c}$ such that $exp(-K_{c})$ would be equal to the probability ranked at round $\left(p*N_{c}\right)$, where $N_{c}$ is the amount of images predicted as class *C*. The maximum output probability for each unlabeled sample was taken in descending order, sorting such probabilities across all samples. *p* is a proportion scaled between [0,1], and optimizing the pseudo-labels produced the p×100% highest confident pseudo-labeled samples for training of the model. Such a technique takes the probability ranked at p×100% separately from each class to both threshold and normalize the confidence levels. To design the self-paced learning framework that incorporates more pseudo-labels for each iteration, we initialized *p* from the top 20% and reliably added the 5% topmost pseudo-labeled samples in each additional iteration of generating and selecting pseudo-labels. To ensure that a superior classification accuracy was achieved, we set the maximum portion *p* to 40% of the topmost pseudo-labeled samples for the selection and retraining process.
**Algorithm 1:**Algorithm for determining $K_{c}$.
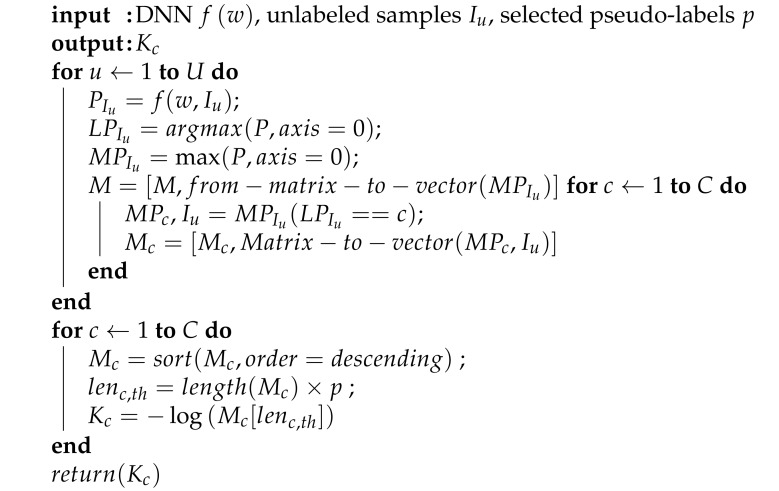


## 3. Experimental Settings

In this section, we perform n extensive evaluation of the proposed method (ROSST) by conducting experiments on two traffic sign recognition benchmark datasets. We firstly explore the contributions of each proposed module and then go ahead to compare our results with the state-of-the-art methods.

### 3.1. Datasets

Most researches and methods have been evaluated on one of these publicly available datasets with a relatively small amount of traffic sign categories.

- The German Traffic-Sign Recognition Benchmark (GTSRB) [3]: It has 43 classes of traffic signs, and it is intended for recognition and classification tasks only. It is made up of tiny images that have been collected from several cities in Germany. The total number of traffic signs in GTSRB is 51,839: 12,630 images for testing and 39,209 images for training. Just like German Traffic-Sign Detection Benchmark (GTSDB), it consists of three super-categories.
- The Belgium Traffic Signs (BTSC) dataset [47]: It is a variation of GTSRB, but has the number of categories extended from 43 to 62. It is designed for traffic sign recognition only. The images are samples of signs used by motorists and pedestrians in Belgium.

The GTSRB and BTSC are publicly available datasets that allow unbiased comparison of various methods for traffic sign recognition, and many of these methods have achieved high recognition accuracy on the GTSRB. The signs have been designed with regular shapes such as circles, triangles, and rectangles, conspicuous colors to attract human drivers’ attention, and positioned at places that can easily be seen by human drivers. However, there are many difficulties in identifying traffic signs by computer algorithms due to illumination variations, color deterioration, blurring from motion, cluttered and scattered backgrounds, and partial occlusions, as shown in Figure 1. The GTSRB and BTSC are datasets that present us with the various challenges that face computer algorithms in obtaining high recognition accuracy in the absence of sample annotations, and the proposed method was evaluated on the two traffic sign recognition datasets with a summary of the specific information of each set provided in Table 1. Since the proposed scheme is a semi-supervised learning approach, the GTSRB training set was divided with a 60:40 percent ratio; 40% was added to the test set to be used as the unlabeled data, and the remaining 60% were divided into 70% and 30% for training and validating the network, respectively. Furthermore, for the BSTC training set, the splitting ratio was 50% for training and 50% for validation. The test set was designated as the unlabeled data. The test sets of both datasets were designated as unlabeled data because their ground truth labels were dropped and not utilized at evaluation time, as well as updating the model at each iteration. The image sizes of the traffic signs varied from 15×15 pixels to 222×193 pixels, which are characterized by many similarities among classes, occlusion, intra-class variations, a great deal of imbalance, and lighting and had low contrast, making it challenging for even humans to sometimes recognize. We explored each proposed module of ROSST on these difficult datasets and show the comprehensive results on each unit.

### 3.2. Network Protocol and Experiment Settings

**Figure A1:**
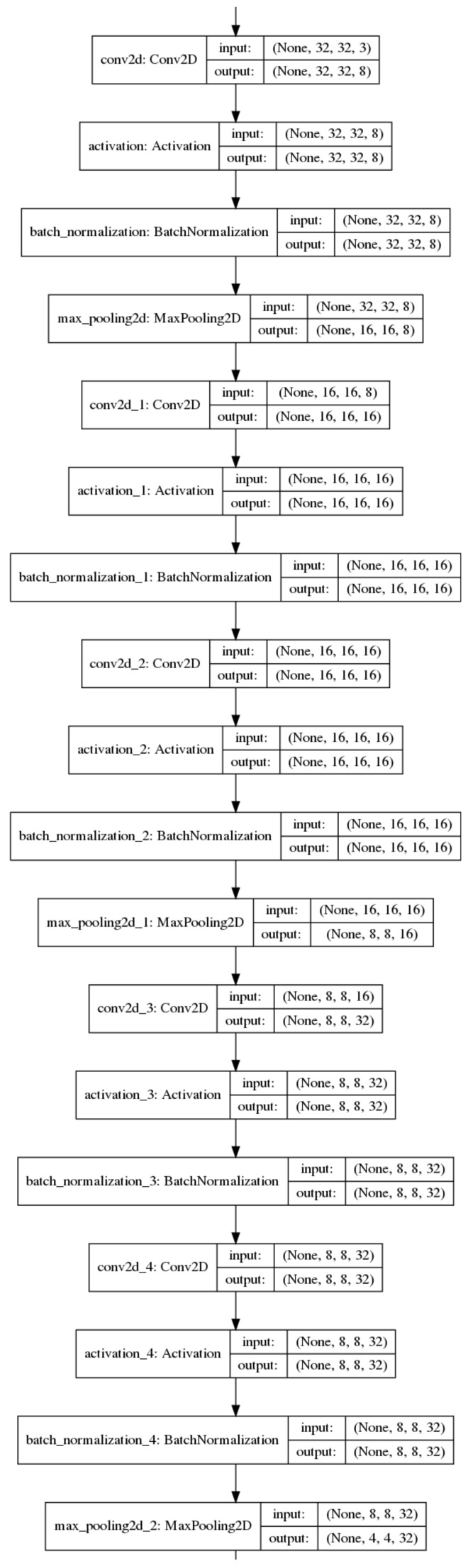
The details of the CNNs in our method.

In the following experiments, a custom network as shown in Figure A1 in Appendix A was built and trained from scratch to serve as the backbone protocol. For both TSR and the Class Activation Map (CAM), there were five convolutional layers and batch normalization layers and three pooling layers that were sequentially stacked. Rectified linear unit activation was used after each convolutional layer. The initial set of layers used a 5×5 kernel to learn larger features to distinguish between different sign shapes and color blobs.

**Figure A2:**
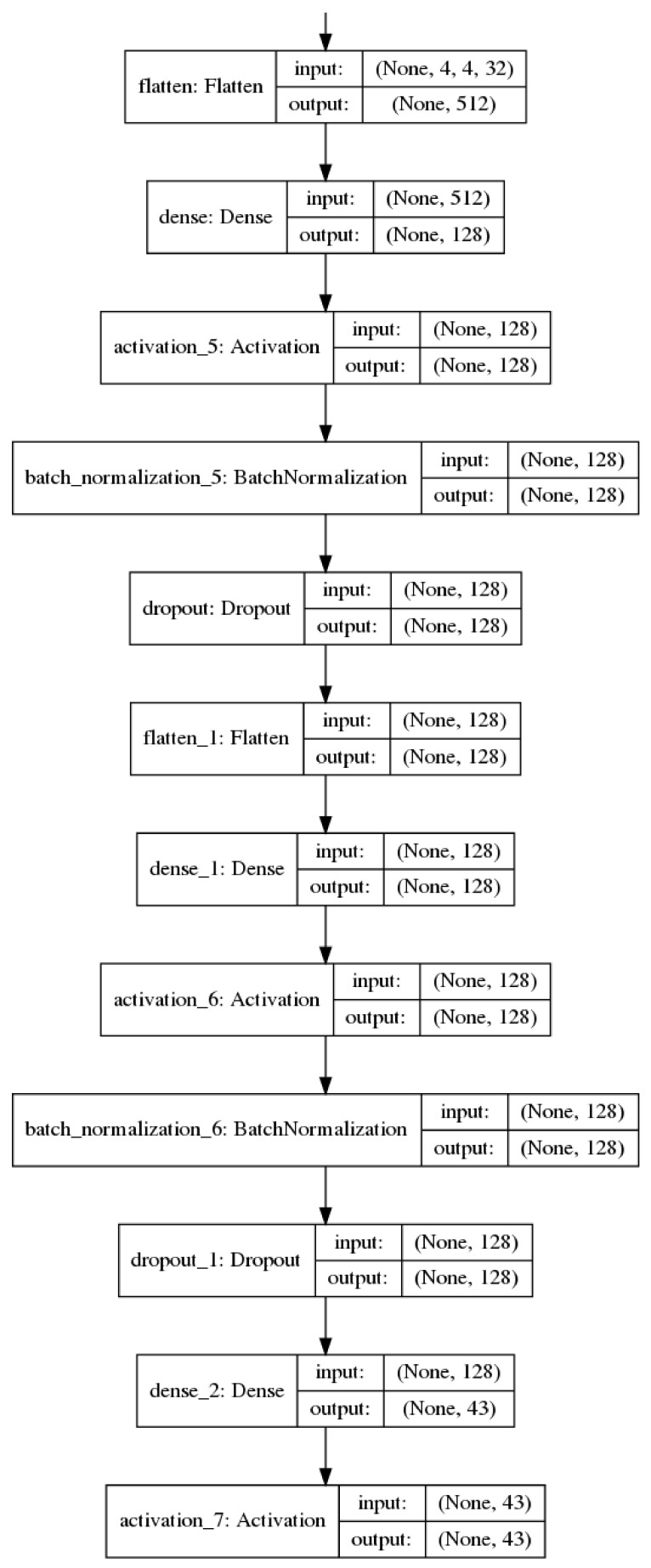
Fully-connected layers for classification.

The TSR-CNN used fully-connected layers and a softmax classifier to produce the final classification result as depicted in Figure A2, whereas the WSL used a convolutional layer having a 1×1 kernel to obtain the attention maps, which would be cropped later to augment the training data. For the feature pooling function g(·), we adopted Global Average Pooling (GAP), and the threshold for the attention cropping was set to 0.5, as given in [39]. Since the images had varying sizes, we resized the images to 32×32 before convoluting them. In this way, we ensured that the input of the CNN was fixed to 32×32.

In the fully-supervised learning phase, we trained the model by utilizing the Adam optimizer [48] with $\beta_{1}=0.9,\;\beta_{2}=0.999$, 50 epochs to train, a mini-batch of 64, and an initial learning rate of 1×10${}^{-3}$, which decayed upon countering a plateau. The same was used for the weak attention learning training procedure. An NVIDIA GTX1080Ti GPU was used to conduct the experiments and retrain our ROSST model with a hyper-parameter for $K_{c}$ pseudo-labeled samples of the unlabeled data, in all the experiments that we conducted. Although it may be argued that fine-tuning of pre-trained networks using ImageNet, which has been evaluated in-depth by various studies and has been show, to be among the best techniques for deep CNNs to improve performance when applied to small data problems, we stand by the fact that such a technique would always obtain near perfect performance since the ImageNet dataset constitutes most if not all of the traffic signs in the benchmark datasets. Thereby, we trained a customized network to learn a model from end-to-end with random network initialization. Data augmentation of rotation, width, and height shifting techniques was performed to regularize the model. Horizontal flips, vertical flips, zooming, and random cropping techniques were not used, because, in the wild, traffic signs are not flipped. We utilized the attention cropping technique in place of the zooming and random cropping techniques, as more accurate object locations would be provided to the model. The number of iterations for the semi-supervised training phase for this article was five. In conducting the experiments, the evaluation metrics employed to evaluate the model were classification accuracy, F1-score, precision, and recall. The classification accuracy was the commonly used evaluation metric to evaluate most of the classification algorithms mentioned in the literature [1,16,30,31]. In simple terms, precision denotes the percentage of relevant samples among the retrieved samples and recall the percentage of relevant samples retrieved over the total relevant samples. The F1-score combines the precision and recall of two indicators and evaluates the overall performance of the classifier. From the evaluation results presented both in the figures and tables, it can be said that the proposed model was more robust in being able to classify objects with improved recognition rates. The time consumed from training the network model to testing for the baseline model lasted for approximately two and half hours (2 h 28 min), but the semi-supervised phase took much more training time (approximately 7 h) for the five iterations of self-training.

## 4. Results and Discussion

By the description provided in Section 2, the proposed ROSST chiefly consisted of three modules, which consisted of weakly-supervised learning with a focus on attention mapping and cropping, pseudo-labels’ generation and selection $\left(K_{c}\right)$, and self-training with self-paced learning. For the sake of reporting, the proposed model was experimented on the GTSRB dataset to demonstrate how each component helped to improve the recognition rate, as shown in Table 2.

For the first proposed module, from Figure 3, we visualized the images by class activation maps on the GTSRB dataset. It can be seen that the model accurately suggested the object part from which it was learning from. The first row contains the raw input image samples that were fed into the model; the second and third rows give the attention maps of the locations of the traffic signs that were accurately predicted; and the fourth row has the bounding box of the most localized part to be cropped and enlarged to augment the training set. For clarity, the attention map was obtained and augmented to the training set for only the first stage, which was the supervised learning phase when the experiments were conducted. The second module, which involved the generation and selection scheme of pseudo-labels using $K_{c}$, as shown in a previous section, was a crucial factor that controlled and determined the amount of pseudo-labeled samples to be selected and used to update the model in each iteration. The third component was the semi-supervised training with a self-paced learning technique to learn and optimize the model jointly; thus making sure that hard-to-transfer samples, which were the least prediction classes according to the unlabeled data prediction portions, that would be ignored were not rejected because of the selection scheme.

**Figure 4:**
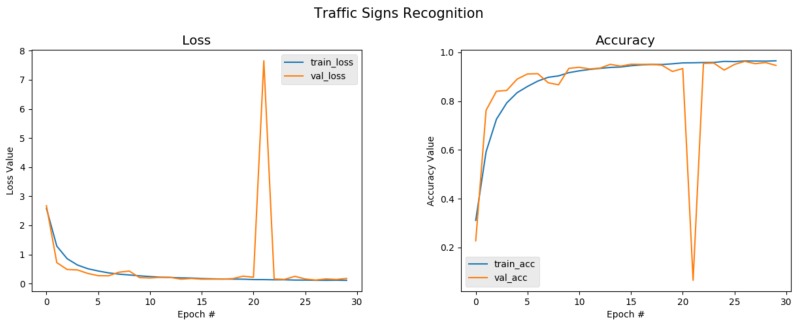
Baseline accuracy and loss plot on GTSRB: The baseline classifier obtains a little over 95% accuracy with no cropped attention maps being used. However, just after Epoch Number 20, it can be seen that there was a sharp drop. Furthermore, it can be observed that the accuracy obtained was below the state-of-the-art accuracy, and this can be attributed to training the network from scratch; however, this was not the case when the semi-supervised scheme was implemented.

**Figure 5:**
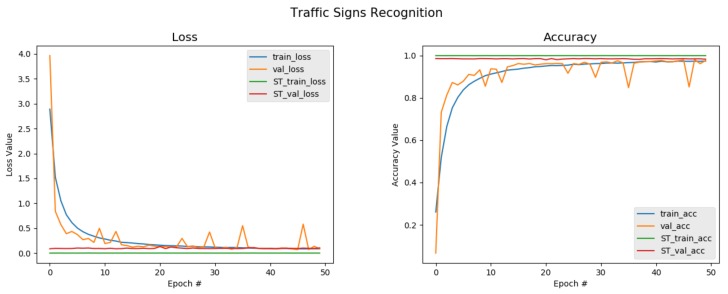
Accuracy and loss plot on GTSRB: The plot has fewer shorter spikes and is smoother compared to the plot in Figure 4 when the cropped attention maps are added to the training set. It can be seen that the classification accuracy for the self-training from the figure is higher than the baseline. This shows that the proposed method has the capacity to classify traffic signs just as the supervised learning schemes.

From Table 2, we can observe that the model with all three proposed modules implemented achieved a greater performance than the others that had either two, one, or none of the modules implemented. It can clearly be seen that the classifier obtained the highest classification accuracy, F1-score, precision, and recall rates of 99.27%, 99.94%, 99.97%, and 99.93%, respectively. This meant that each component was very vital as far as improving the recognition rates using a semi-supervised learning approach on a small set of data was concerned. The relationships between the loss, accuracy, and trained epochs of the networks are shown in Figure 4 and Figure 5 for both the baseline model and the self-training model, respectively. For the baseline, the classification accuracy maintained a substantial increase, but dropped sharply just after the 20th epoch and picked up afterwards to gain a classification accuracy of 95.43%, which was quite decent considering the fact that the network was trained from scratch with random initialization. In particular, the baseline network was trained on the entire training set with a ratio of 70:30 for training and validating, respectively, and then testing the network model using the test set to obtain that recognition rate. Likewise, the loss showed an obvious decline as the number of epochs increased due to the training before the first 20 epochs, but suddenly rocketed very high and later dropped to a very low mark. When compared to the classification accuracy reported in the literature, the baseline accuracy was far below. This was due to the fact that the baseline CNN lacked enough training data, although data augmentation was implemented, a big challenge facing deep neural networks. Figure 5, however, changes the narrative.

**Figure 6:**
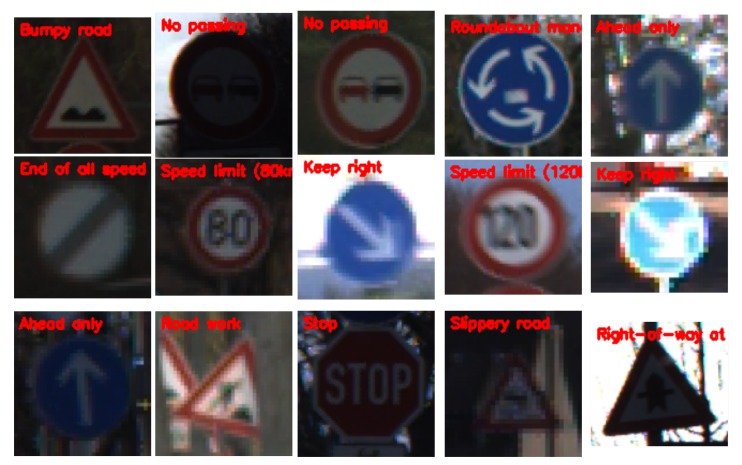
Prediction on sampled web images: The model achieved a perfect performance in classifying image samples from the web.

**Figure 7:**
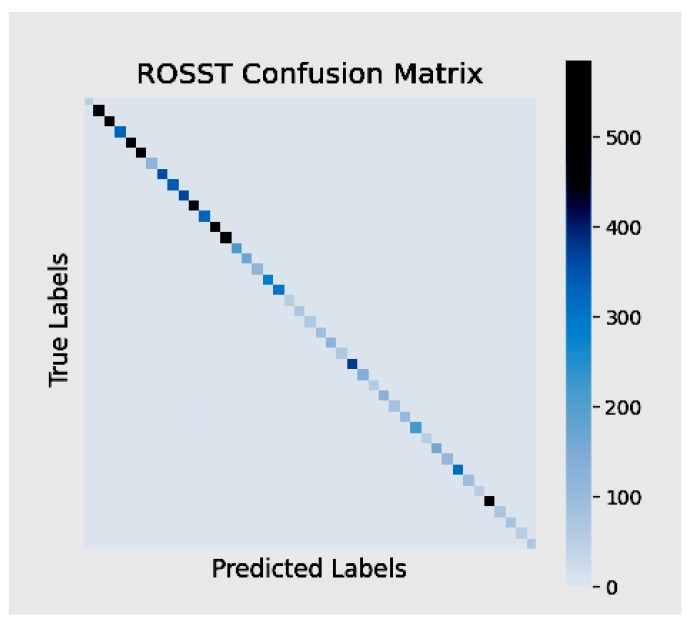
ROSST confusion matrix.

Looking at Figure 5, it can be seen that the classification accuracy steadily increased with no issue of sharply dropping or over-fitting. Although the training accuracy learning curve smoothly increased, the validation curve was not so smooth. It could also be seen that the validation curve had spikes. Yet, it was able to obtain a better performance, comparing it with the baseline, where the entire training set was used, and in this scenario, only 60% of the training set was used to train the classifier. It can be further observed that the proposed self-training scheme improved all the performance by substantially maintaining a classification accuracy of 99.27%, and this can be attributed to the three key components of attention cropping, the pseudo-label generation, and the selection technique, as well as utilizing $K_{c}$ in the self-training scheme to learn from easy-to-hard samples. The classification accuracy of the self-training model maintained a stable value, and the value of the loss of the model was the least obtained. Therefore, we assert that with our algorithm, the recognition rate reached after training was comparable to some state-of-the-art models that implemented fully-supervised learning algorithms. To test the model, we evaluated the model on images copied from the web and made the model predict and tag the images with to which classes they may belong. From the visualization provided in Figure 6, it was observed that the model predicted the classes and labels on the images very accurately. This has not seen before, which goes to prove the robustness and efficiency of the proposed model. To further evaluate the efficacy of the proposed algorithm, a confusion matrix was generated to see if the model had difficulties in predicting the labels for the various classes. Figure 7 depicts the confusion matrix, and from it, it can be observed that the model had no confusion at all when it came to classifying the traffic signs.

The result in Table 3 provides a comparison of the classification accuracy between ROSST, the proposed method, and some state-of-the-art supervised learning algorithms, which include single CNN with three STNs [29], DCGAN-PILAE [30], traffic sign classification based on pLSA [16], multiscale CNNs [31], BAGAN [32], traffic sign recognition with hinge loss CNNs (HLSGD) [49], and residual blocks CNN [50]. All these state-of-the-art methods were evaluated on the GTSRB dataset, making it fair to compare the proposed method ROSST with them. It is essential to note that, although the proposed method used a semi-supervised technique, it obtained an accuracy of 99.27%, which surpassed the BAGAN, pLSA, and multiscale CNN algorithms by over 2%, 1.03%, and 0.33%, respectively. The ROSST’s recognition rate however trailed the other supervised learning methods with a smaller margin. This occurred because those frameworks had accuracy rates that were higher than the proposed method.

The state-of-the-art models [29,30,49,50] used all of the training set to train the model and then were tested on the test set, but the proposed method used 60% of the training data to train and validate the model during the supervised training stage and later ran on the unlabeled set(the remainder of training plus the test sets) for the self-paced learning phase. This showed that the model could perform well and achieve a higher recognition rate on a small set of data and train a good model just as the state-of-the-art models.

To compare the proposed approach with some state-of-the-art semi-supervised algorithms that were evaluated on the GTSRB including TSCA co-training [1] and multiple feature representation [46], TSCA co-training [1] proposed by Hillebrand et al. was trained on just 14 classes. A recognition accuracy of 99.71% was obtained for precision, recall, and F1-score for the GTSRB and for the BTSC, 98.95%, 98.87%, 98.86%, respectively. German street traffic signs are similar to the benchmark dataset GTSRB, which has 43 classes. It was therefore fair to do a comparison between the proposed method’s (ROSST) accuracy and TSCA co-training’s recognition accuracy. It can be seen from Table 4 that the proposed method’s classification accuracy was higher than all the methods. For multiple feature representation [46], the authors proposed two techniques and evaluated them on the GTSRB to obtain 98.59% and 98.77% accuracy after using 50% of the training set against the proposed model, which used a 20% ratio of the labeled training set and yet managed to obtain the topmost classification accuracy.

We further evaluated the proposed ROSST on the BTSC dataset, and in this way, we obtained a viable model that performed effectively on arbitrary data. Since the BTSC is a dataset that is much smaller than the GTSRB, we split the training set into a 50 percent training set and 50 percent validation set, respectively. The test set was designated as the unlabeled data, where the labels were dropped and the generated pseudo-labels were rather used to update the model in each iteration. Our proposed method achieved a recognition rate of 98.97% when evaluated on the BTSC, and as is shown in Table 5, the accuracy was just little below the state-of-the-art methods. The proposed algorithm, which was combined as part of the self-training process, was helpful, and even in the face of supervised learning methods, the proposed method could still manage a decent score that was not even up to a margin of 1%. Performance results like the ones reported in Table 4 and Table 5 demonstrated that our method achieved not only an outstanding classification accuracy, but also showed robustness on the two European traffic sign recognition datasets.

### Accuracy of $K_{c}$ with Different Ratios of Pseudo-Labeled Samples

Table 6 provides the various classification accuracies for the ratios of pseudo-labeled data selected to update the model in further iterations. From the table, $K_{c}$ was set to 10%, 20%, 30%, 40%, 50%, and 100% of the amount of pseudo-labeled samples selected from the generated pseudo-labels of the entire set and from each class to update the training set that would be used to train the model in the next iteration, and this met the condition of the small sample problem. The bolded accuracy points out the highest accuracy in the results column. For example, in the third column of Table 6, where the proportion of $K_{c}$ was 20% of the generated pseudo-labeled samples, the self-training strategy had the highest accuracy of 99.27% on the GTSRB. In column 5, where the ratio of the selected pseudo-labeled samples was 40%, an accuracy of 98.97% was obtained when the model was evaluated on the BTSC dataset. One major observation was that, for the GTSRB, apart from obtaining the best accuracy when $K_{c}=20\%$, the results for the other ratios were not that great. To find out why the model failed to match the result obtained with $K_{c}=20\%$, it was realized that the bigger the portions were, the more the model struggled to obtain a good classification accuracy, and this is evident in Table 6 where $K_{c}=40\%,50\%$, and 100%. It was also realized that for $K_{c}=10\%$, the model achieved the lowest recognition rate, and this could be attributed to the lack of enough training samples. Similar observations were made for the BTSC, where $K_{c}=50\%$ and 100%. These results showed that the proposed strategy fully considered useful information, which was accessible to guide the learning process, as well as improve the recognition rate as far as the small amount of labeled data was concerned. To further prove the effectiveness of our method, comparison experiments with our strategy and other fully-supervised learning algorithms and semi-supervised learning methods evaluated on both the GTSRB and BTSC were carried out for the sake of fairness. The accuracy of other methods came from the corresponding references. As shown by the classification accuracy in Table 4 and Table 5, the accuracy of our method was very close to that of the fully-supervised learning algorithms and far surpassed the semi-supervised learning methods, which proved that the proposed self-training approach was able to handle the small sample problem on the GTSRB and BTSC datasets.

## 5. Conclusions

Challenges such as poor image quality due to low resolution, bad weather conditions, illumination, either above or below, occlusion, and deterioration of the traffic signs, coupled with a lack of sufficient labeled data, make it a daunting task for computer algorithms to recognize and determine the categories of traffic signs. To overcome these challenges, we proposed a robust semi-supervised learning (ROSST) framework for traffic sign recognition, especially for tasks with an insufficient amount of labeled data and imbalanced data. The proposed approach introduced weakly-supervised learning to map discriminative parts and augment the training set with reliable extracted parts of image samples. A self-paced learning scheme was then introduced to correct and mitigate reinforcing the wrongly generated pseudo-labels for unlabeled data in enlarging the training set before the model was updated in the next iteration. We proposed a novel and efficient sample selection algorithm that mitigated the problems of conventional self-training methods, such as: holding onto better represented and easy-to-transfer class samples; ignoring less represented samples in the pseudo-label generation and selection procedure for imbalanced data; and mistakenly reinforcing incorrect pseudo-labeled samples to retrain the model. The results of the extensive experiments conducted clearly showed that the proposed method was robust and that the classification accuracy of our approach surpassed the accuracy of other semi-supervised algorithms, even obtaining results close to the state-of-the-art supervised learning algorithms when evaluated on the GTSRB and BTSC. The experiments were designed to utilize 60% and 50% of the labeled training set for both the GTSRB and BTSC, respectively. In this way, it met the condition of the small-sample problem. Therefore, we seek to further investigate how to improve the generalization of our approach to a more difficult task like Google Street View, which can be widely used in many countries.

## Figures and Tables

**Table 1:** A summary of the information of the state-of-the-art benchmarks.

Dataset	# of Classes	# for Training	# for Testing
GTSRB	43	39,209	12,630
BTSC	62	4637	2534

**Table 2:** Proposed contributing components and their combinations. $K_{c}$ is the pseudo-label selection component.

Attention Cropping	$K_{c}$	Self-Training	Acc (%)	F1 (%)	Precision (%)	Recall (%)
			95.43	94.07	95.71	94.01
🗸			96.57	95.84	97.31	95.91
🗸		🗸	97.09	98.02	96.95	96.63
🗸	🗸	🗸	**99.27**	**99.94**	**99.97**	**99.93**

**Table 3:** Comparison of the classification accuracy of our approach and the state-of-the-art supervised algorithms on the GTSRB.

Methods	Acc (%)
Single CNN with 3 STNs [29]	99.71
DCGAN-PILAE [30]	**99.80**
pLSA [16]	98.14
Multiscale CNNs [31]	98.84
BAGAN [32]	96.75
HLSGD [49]	99.65
Residual blocks CNN [50]	99.33
ROSST (our approach)	99.27

**Table 4:** Comparison of the classification accuracy of our approach and some semi-supervised methods on the GTSRB.

Methods	Acc (%)
Multiple feature representation (parallel fusion) [46]	98.59
Multiple feature representation (serial fusion) [46]	98.77
TSCAco-training (14 classes) [1]	98.00
ROSST (our approach)	**99.27**

**Table 5:** Comparison of the classification accuracy of our approach and some supervised methods on the BTSC.

Methods	Acc (%)
Residual blocks CNN [50]	99.17
Single CNN with 3 STNs [29]	99.71
DCGAN-PILAE [30]	**99.80**
VGG-16 [51]	99.72
ROSST (our approach)	98.97

**Table 6:** Accuracy of traffic sign datasets for $K_{c}$ with different proportions of pseudo-labeled samples.

Proportions ($K_{c}$)	10%	20%	30%	40%	50%	100%
BTSC	95.76	97.37	94.92	**98.97**	98.12	96.68
GTSRB	96.83	**99.27**	98.96	97.43	98.29	97.94

## Abbreviations

The following abbreviations are used in this manuscript:

ROSST	Robust Semi-Supervised Traffic Sign Recognition
DNN	Deep Neural Network
CNN	Convolutional Neural Network
GTSRB	German Traffic-Sign Recognition Benchmark
BTSC	Belgium Traffic Sign for Classification dataset
WSL	Weakly-Supervised Learning
SPL	Self-Paced Learning

## Appendix A. Network Architecture

Figure A1 and Figure A2 provide the layout of the network architecture. The architecture consists of sequentially stacked convolutional layers, pooling layers, normalization layers, and activation layers.
